# Supplementary material for: MetaRibo-Seq measures translation in microbiomes
Source: Nat Commun. 2020 Jun 29;11:3268. doi: 10.1038/s41467-020-17081-z (PMC7324362; doi:10.1038/s41467-020-17081-z)
Supplement: Supplementary file 10 — Supplementary Data 7 [file 41467_2020_17081_MOESM10_ESM.zip › File2/Confidence_VeryHigh_Taxonomy/10356_out.krona.html]

Javascript must be enabled to view this page.

members
magnitude
magnitudeUnassigned
count
unassigned
taxon
rank

10356\_out

4

superkingdom
2
4

4
phylum
1239

4
class
186801

4
186802
order

4
family
31979

1649459
genus
4

species
154046

SRS019601\_contig\_number\_12804SRS019601\_contig\_number\_29690SRS019601\_contig\_number\_contig-100\_2789.171810SRS146888\_contig\_number\_13512
4
